# The early immune response to infection of chickens with Infectious Bronchitis Virus (IBV) in susceptible and resistant birds

**DOI:** 10.1186/s12917-015-0575-6

**Published:** 2015-10-09

**Authors:** Jacqueline Smith, Jean-Remy Sadeyen, David Cavanagh, Pete Kaiser, David W. Burt

**Affiliations:** The Roslin Institute & R(D)SVS, University of Edinburgh, Easter Bush, Midlothian, EH25 9RG UK; The Pirbright Institute, Compton Laboratory, Compton, Berkshire RG20 7NN UK

**Keywords:** Chicken, Infectious bronchitis virus, Microarray, Disease resistance, Candidate gene

## Abstract

**Background:**

Infectious Bronchitis is a highly contagious respiratory disease which causes tracheal lesions and also affects the reproductive tract and is responsible for large economic losses to the poultry industry every year. This is due to both mortality (either directly provoked by IBV itself or due to subsequent bacterial infection) and lost egg production. The virus is difficult to control by vaccination, so new methods to curb the impact of the disease need to be sought. Here, we seek to identify genes conferring resistance to this coronavirus, which could help in selective breeding programs to rear chickens which do not succumb to the effects of this disease.

**Methods:**

Whole genome gene expression microarrays were used to analyse the gene expression differences, which occur upon infection of birds with Infectious Bronchitis Virus (IBV). Tracheal tissue was examined from control and infected birds at 2, 3 and 4 days post-infection in birds known to be either susceptible or resistant to the virus. The host innate immune response was evaluated over these 3 days and differences between the susceptible and resistant lines examined.

**Results:**

Genes and biological pathways involved in the early host response to IBV infection were determined andgene expression differences between susceptible and resistant birds were identified. Potential candidate genes for resistance to IBV are highlighted.

**Conclusions:**

The early host response to IBV is analysed and potential candidate genes for disease resistance are identified. These putative resistance genes can be used as targets for future genetic and functional studies to prove a causative link with resistance to IBV.

**Electronic supplementary material:**

The online version of this article (doi:10.1186/s12917-015-0575-6) contains supplementary material, which is available to authorized users.

## Background

Infectious bronchitis (IB) is a highly contagious respiratory disease of chickens first described in the USA in the 1930’s [1–3]. Clinical signs include: coughing, sneezing, rales and nasal discharge. The disease can also affect the reproductive organs, which leads to a decrease in egg quality and production, thus making it a major cause of economic losses within the poultry industry [4]. The causative virus, Infectious Bronchitis Virus (IBV) is a coronavirus, which is an enveloped virus with a single positive-stranded RNA genome, which replicates in the host cell cytoplasm [5]. Proteins encoded by IBV include the viral RNA polymerase, structural spike proteins, membrane and nucleocapsid and various other regulatory proteins. The spike glycoprotein mediates cell attachment and plays a significant role in host cell specificity [6].

The existence of many different IBV serotypes, which are not cross-protective means that control of IB, is very difficult. Mortality is usually fairly low (~5 %), however some strains of the virus can also cause nephritis meaning that, depending on strain, mortality can be greater than 50 % [7, 8] or even up to 80 % with some Australian isolates [9]. IBV infection leaves birds more susceptible to colibacillosis [10] and subsequent bacterial infections can also lead to a high level of mortality [11]. Currently, attenuated live vaccines are used in broilers and pullets, and killed vaccines are used in layers and breeders [12]. However, virus control is very difficult, as there are only a few vaccine types and many different strains of IBV. The virus also continues to mutate rapidly, generating more virulent strains of the disease [13–15]. Coronaviruses have now also been detected in other avian species such as turkey, duck, goose, pheasant, guinea fowl, teal, pigeon, peafowl and partridge [4].

The extent to which the virus affects the host is highly dependent on the chicken breed [4] and the MHC B locus is known to play a role in susceptibility to the virus [16]. In this study we attempt to identify non-MHC genes, which may be involved in resistance to IBV. No genetic analyses have thus far been undertaken in order to try and do this and no quantitative trait loci or genes associated with resistance have been determined, so far. Based on differential gene expression in susceptible and resistant lines of chickens, we identify potential candidate genes for disease resistance towards IBV (virulent M41 strain). Building on the previous work by Dar et al. [17] and Wang et al. [18] we used Affymetrix whole-genome chicken microarrays to examine the tracheal gene expression profiles of a line of birds known to be susceptible to IBV infection (line 15I) and a line known to show resistance (line N). We determined the early host response to infection and propose possible candidate genes for involvement in disease resistance towards IBV. Understanding how coronaviruses infect the host and identifying genes involved in resistance is important not only for the poultry industry but also has important implications for human health, as diseases such as SARS are also caused by coronaviruses [19, 20].

## Methods

### Ethics statement

All animal work was conducted according to UK Home Office guidelines and approved by the Roslin Institute Animal Welfare and Ethical Review Body.

### Experimental animals

The lines used in these experiments are an IBV susceptible line – line 15I (inbred White Leghorn strain) [21] and an IBV resistant line – line N (non-inbred Cornell strain). Line 15I was developed at East Lansing in the USA in the 1940s [22] and Line N at Cornell, USA in the 1960s [23]. The lines have since been maintained at the Institute for Animal Health in Compton, UK. Two-week-old chicks from each line (15I and N) were separated into two experimental rooms, with *ad libitum* access to food and water. In one room, 54 birds (27 from each line) were infected with 4 log_10_ CID_50_ (10^4^ CID_50_) of virulent IBV-M41 strain in a total of 100 μl of 0.2 % BSA in PBS equally by intra nasal and ocular routes. In the other room, 54 control birds (27 from each line) received 100ul PBS via the same route. Trachea samples (upper half) were collected at 2, 3 and 4 days post-infection (9 individual birds from each line at each time point). The trachea of infected and control birds from each line were analysed for viral load using Taqman real-time quantitative RT-PCR assays.

### RNA preparation

Tissue samples (~30 mg) were stabilized in RNAlater (Ambion, Life Technologies, Paisley, UK) and disrupted using a bead mill (Retsch MM 300, Retsch, Haan, Germany) at 20 Hz for 4 min. Total RNA was prepared using an RNeasy kit (Qiagen, Crawley, UK) extraction method as per the manufacturer’s protocol. Samples were resuspended in a final volume of 50 μl of RNAse-free water. Concentrations of the samples were calculated by measuring OD_260_ and OD_280_ on a spectrophotometer (Nanodrop, Thermo Scientific, Paisley, UK). Quality of the RNA was checked on a bioanalyser (Agilent Technologies, South Queensferry, UK). An RNA integrity number (RIN) > 8 proved the integrity of the RNA.

### Whole genome gene expression microarray hybridization

Biotinylated fragmented cRNA was hybridized to the Affymetrix Chicken Genome Array. This array contains comprehensive coverage of 32,773 transcripts corresponding to over 28,000 chicken genes. The Chicken Genome Array also contains 689 probe sets for detecting 684 transcripts from 17 avian viruses. For each experimental group (control and infected birds in each of the two lines at each of 2, 3 and 4 dpi), three biological replicates (3 RNA pools from 3 birds) were hybridized. Thus, 36 arrays were used in total. Hybridization was performed at 45 °C for 16 hours in a hybridization oven with constant rotation (60 rpm). The microarrays were then automatically washed and stained with streptavidin-phycoerythrin conjugate (SAPE; Invitrogen, Paisley, UK) in a Genechip Fluidics Station (Affymetrix, Santa Clara, CA). Fluorescence intensities were scanned with a GeneArray Scanner 3000 (Affymetrix, Santa Clara, CA). The scanned images were inspected and analyzed using established quality control measures. Array data have been submitted to Array Express (http://www.ebi.ac.uk/arrayexpress/) under the Accession Number E-TABM-1128.

### Statistical analysis

Gene expression data generated from the GeneChip Operating Software (GCOS) was normalised using the PLIER (probe logarithmic intensity error) method [24] within the Affymetrix Expression Console software package. This normalised data was then analysed using the limma and FARMS [25] packages within R in Bioconductor [26]. Probes with a False Discovery Rate (FDR) value <0.05 and a fold change ≥1.5 were deemed to be biologically significant.

### Analysis of differentially-expressed genes

In order to determine which biological pathways are involved in the responses to viral infection, we analysed our differentially-expressed (DE) genes using Pathway Express [27, 28] which uses KEGG pathways [29] to pictorially display up/down regulation of genes. (NB. These diagrams are based on the human pathways and so are not completely representative of the chicken pathways). Genes differentially expressed during the host response (FDR <0.05) were analysed against a reference background consisting of all genes expressed in the experiment. Factors considered by Pathway Express include the magnitude of a gene’s expression change and its position and interactions in any given pathway, thus including an ‘impact factor’ when calculating statistically significant pathways. Anything with a *p*-value <0.25 is deemed significant when using this software. Use of the Ingenuity Pathway Analysis (IPA) program [30] revealed which canonical pathways are being switched on by IBV infection in the host (with Benjamini-Hochberg multiple testing correction) and allowed us to analyze the gene interaction networks involved in the host response. Genes were clustered by similar expression pattern and analysed for enriched GO-terms and transcription factor binding sites (TFBS) using Expander (v5.2) [31]. Normalised expression data from control samples were compared with infected samples to examine the host response to IBV infection. Enrichment analysis of particular GO terms or TFBS within clusters was done using the TANGO and PRIMA functions, respectively, within the Expander package.

### Viral quantification and specific gene expression analysis by quantitative real-time PCR

Taqman real-time quantitative RT-PCR (qRT-PCR) was used to quantify viral RNA levels and for confirmation of the microarray results for the mRNA levels of selected genes. This was performed on 3 replicate pools of 3 samples (9 birds). Primers (Sigma) and probe (PE Applied Biosystems, Warrington, UK) (Table 1) were designed using Primer Express (PE Applied Biosystems). Briefly, the assays were performed using 2 μL of total RNA and the Taqman FAST Universal PCR Master Mix and one-step RT-PCR mastermix reagents (PE Applied Biosystems) in a 10 μL reaction. Amplification and detection of specific products were performed using the Applied Biosystems 7500 Fast Real-Time PCR System with the following cycle profile: one cycle at 48 °C for 30 min and 95 °C for 20 sec, followed by 40 cycles at 95 °C for 3 sec and 60 °C for 30 sec. Data are expressed in terms of the cycle threshold (Ct) value, normalised for each sample using the Ct value of 28S rRNA product for the same sample, as well described previously [32–34]. Final results are shown as 40-Ct using the normalised value, or as fold-change from uninfected controls.

**Table 1:** Primers used in QRT-PCR analysis

Gene	Forward primer	Reverse primer	Probe primer	Opt. primer conc.	Probe (μM)	GenBank
28S	GGCGAAGCCAGAGGAAACT	GAC GACCGATTTGCACGTC	AGGACCGCTACGGACCTCCACCA	0.6	5	FM165415
C1S	GCGCAAAGGCTGGAAAATAC	TCAAGAACAGAATTGGGAGTGACA	TACTATGCTGAACCCATAACCTGTCTCCCG	0.6	5	NM_001030777
CCL13	CAGAGCCTGGCCCAGAGA	TGTCCATTTTGATTCTTCTGGTATG	CTGTGCCTGACAAGTGCTGCTTCAACTT	0.2	5	XM_415779
CCLi7 (ah221)	CACAACCTGCTGCTTCTCCTATG	TGTAGGCGGAGGCAATGAG	TCAACGTCCCGTCCCACGCA	0.2	5	AY037860
CD38	GCTTGATGGGCTTTCATGGT	CACATTCACTCCATTTTGGACAA	ACCCCTCAGCTCCAGGAATCAACTATGAA	0.6	5	XM_420774
CLU	TGAGTCAGAATCCCGTAACTTCAG	GCAGTCCACAGCCAAGATCTC	AGATCCGGCGCAACTCGGCC	0.2	5	NM_204900
COX11	TGGGATCTCCACCTACAACGTA	CAGCCACTGTTCTTCAAAACAAA	TGCCCTTCGAAGCAGGACAGTACTTCA	0.4	5	XM_001233972
DDT	GGCCCCGAGCGGATT	CATGACTGTTCTGTTCTTGCCAAT	CATTCGCTTTTACCCGCTGGAGCC	0.8	5	NM_001030667
FK-506-BP51	CGGAGGATCAAGAGGAAAGGA	CAGAACCCCTCCAGGTGAATT	AAGGCTATTCCAACCCCAACGAAGGTG	0.1	5	NM_001005431
HSC20	GGAAATCATGGAAATCAATGAGAAA	CACCTCTTTGGTCAGTTCTTCTTG	CAGAGCCCGAGAACGACGAGATCC	0.8	5	XR_026662
IFNAR2	TGGTCACTGCATCTCTAAATAAACATT	CTGCAATTGTGATGCCATAATAATC	CATCCCATCAGCCTGGAAATGCATAACT	0.4	5	NM_204858
IGFBP5	GAAGAGCAGCCAGAGGATGGT	GCTTGCACTGCTTCCTCTTGT	CACCTCCCCAACTGCGACCGAAA	0.1	5	XM_422069
IRF-7	ACCCGGACCGCCGTAT	GCCCAGGCCTTGAAGATCTC	CATCCCTTGGAAGCACAACGCCA	0.6	5	NM_205372
MAFK	GCGATGATGAACTCGTGTCAA	TTCAGACGGATGACCTCCTCTT	TCCGTACGGGAGCTGAACCAGCAC	0.4	5	NM_204756
MAP4K4	TGCTATTGAAATGGCTGAAGGA	TCCGTGGGATGAGGAAGAGT	TCCTCCCCTGTGTGACATGCACCC	0.2	5	NM_001031126
MMD2	TGCCACGCACGCATTCT	GGTCATCGGAGAGGACGTAGAG	TCCTGCCCAGCATCCTCGGC	0.4	5	XM_414787
MX1	TGGACTTCTGCAACGAATTGTC	ATCCAGAAGAGTGCTGAAATGTTTG	TTCACCTCCGCAATCCAGCAAGAGA	0.6	5	NM_204609
SRI	TACTATCAGGGCGGGTATGGA	AGCAAAATAACCATACAGAGGATCCT	CAGCTCCAGGAGGCCCATCATTCC	0.1	5	NM_001080865
SUCLG2	AGCTTCCCGGCTGTTCAAT	CATGGTCTGCCATCAGCTTTT	AACCCCTAGACGATGGCTGAATCTGCA	0.1	5	NM_001006141
TLR3	ATCCATGGTGCAGGAAGTTTAAG	GATGGAGTCTCGACTTTGCTCAATA	TGCATCATGCTTTACAGC	1.0	5	JF273967.1
TNFAIP1	GTTGTGGGAAGCACTTTGGAA	TCAACTCCTTGATCTCCTGTCTGT	CCGAGATGACACAATTGCACTTCCAAAA	0.2	5	NM_001030726
TP-D53	TGGCCAAGCTAGAGGATGAAA	TCAGGCTCATGCCAAGTTTTT	ACTAGCAGCCAAAGAAAAGCACCTGATTGA	0.2	5	NM_204215

## Results and discussion

### Assessment of viral load

**Fig. 1:**
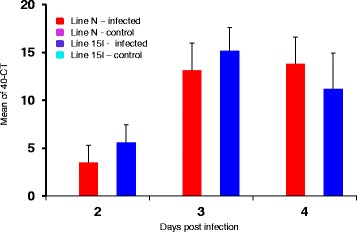
QRT-PCR measurement of viral load in control and infected birds from lines 15I and N. Mean 40-Ct values are shown with the standard error mean indicated

Taqman real-time quantitative RT-PCR analysis was used to measure viral load in trachea samples from both control and infected birds from both lines 15I and N. Tracheal tissue was chosen for examination in this study as the target of IBV is the epithelial surface of the respiratory tract. Viral RNA was detected in infected birds, but no significant difference in viral load was detected between lines at any of the days 2, 3 or 4 post infection (Fig. 1). This would indicate that the resistance to the virus seen in Line N is due to how the birds respond to the virus once it has entered the body and is not a measure of how the birds can prevent initial infection by the virus itself. When resistance to IBV infection was originally determined in these lines, it was noted that they were equally susceptible to infection, but a variation in outcome was seen. In line N, 33 % of birds showed air sac lesions whereas 73 % of 15I birds presented lesions. Mortality was 0 in line N, but 47 % within line 15I birds. It was hypothesized that the different lines were producing different immunological responses upon infection [21].

### Host response to IBV infection

Gene expression differences found in the susceptible 15I line between infected and control birds over days 2, 3 and 4 post infection were analysed, with a view to examining the innate host response to infection by IBV. Genes seen to be induced during the host response to infection include *C1S*, *IRF1*, *STAT1*, *MX1*, *TLR3* and *CTSS* as previously recognised by Guo et al. [35]. We also identified *IFIT5*, *OASL*, *SCA2*, *LYG2*, *ISG12-2*, *DDX60*, *IFIH1*, *IRF7*, *ZC3HAV1*, *DHX58*, *CCli7*, *IFITM1* and *IFITM3* as being up-regulated in response to IBV infection. Few genes are seen to be down-regulated during the early stage of the host response, but those which are include *CHAC1* (pro-apoptotic), *HBB* (implicated in inflammation regulation) and *PDK4* (glucose regulation). For a full list of the genes involved in the tracheal immune response (133 DE probes), see Additional file 1: Table S1.

**Fig. 2:**
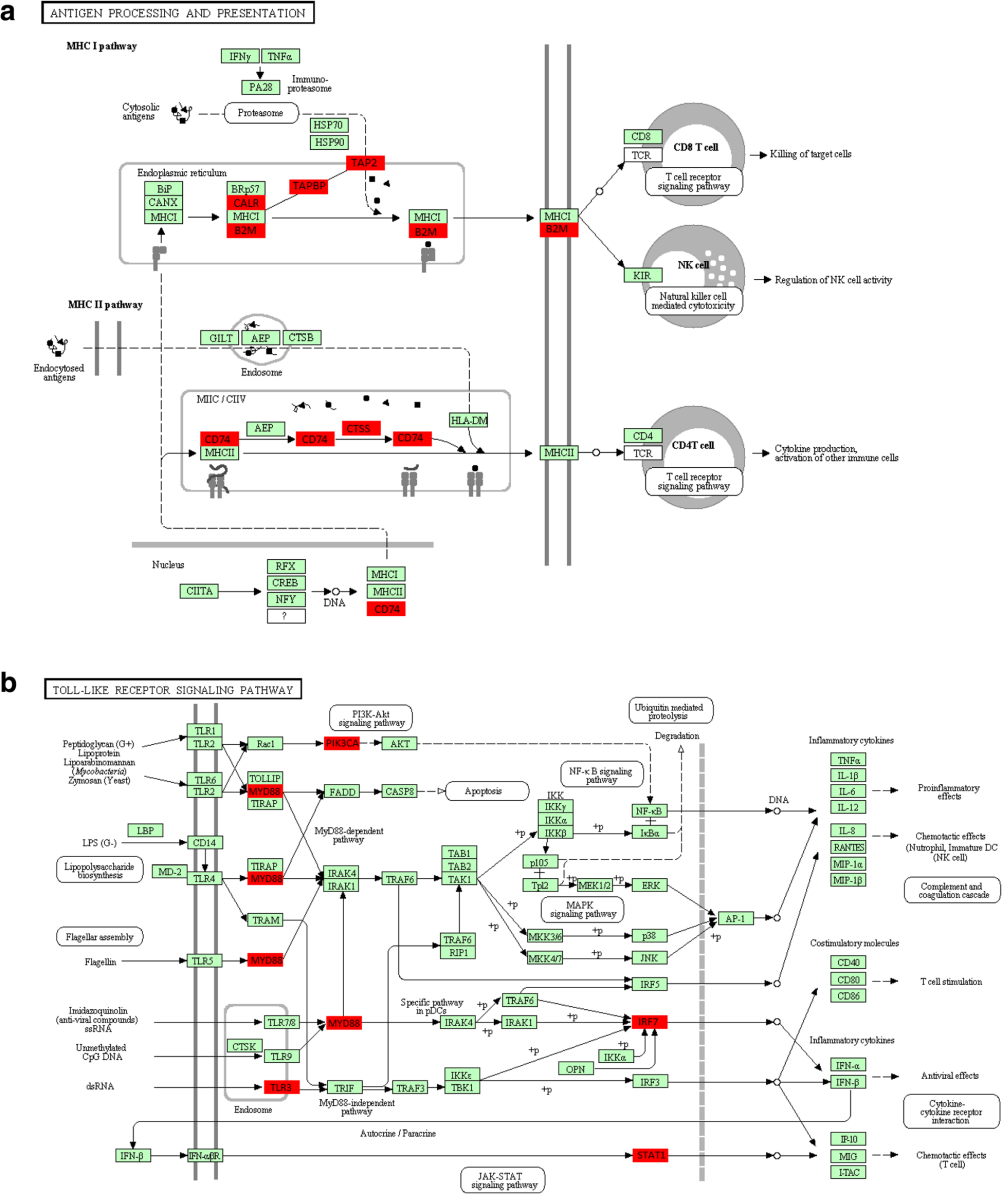
Pathway Express analysis of the host response to IBV infection in the trachea of susceptible birds (Line 15I). Many genes involved in antigen processing and presentation (**a**) and in the Toll-like receptor pathway (**b**) can be seen to be up-regulated (shown in red)

To elucidate which biological pathways are being perturbed during the host response to IBV infection, we analysed our data using Pathway Express [36]. The resulting pathway diagrams are extremely useful in establishing which gene networks are involved in a particular experimental response. As seen in Fig. 2, genes involved in antigen presentation and the Toll-like receptor (TLR) pathway are up-regulated. TLRs identify pathogen associated molecular patterns (PAMPs) and are crucial to the innate immune system. In this study *TLR3* is shown to be induced at 3 dpi. TLR3 recognizes double-stranded RNA intermediates produced during viral replication and has previously been shown to be induced in the trachea at this time after IBV infection [37]. Another pathway involved is the phosphatidylinositol signalling pathway (Table 2). Phosphatidylinositol kinases are known to play an important role in the viral life cycle after infection of the host and PI4KB is known to be exploited by coronaviruses for viral entry. The product of PI4KB catalysis is phosphatidylinositol 4-phosphate (PI4P) and coronavirus entry into the host is mediated by the PI4P lipid microenvironment [38]. Genes involved in the complement system are also highlighted as being up-regulated in response to IBV infection. Complement-mediated lysis of viruses is an important facet of the host innate immune system and its role in defence against viral infection [39] – as reflected in the induction of these genes in this study.

**Table 2:** Pathway Express analysis of the host response to IBV infection

Rank	Pathway name	Impact Factor	Input genes/Genes in Pathway	Corrected gamma *p*-value
1	Antigen processing and presentation	14.244	6/89	9.93E-06
2	Toll-like receptor signaling pathway	7.927	5/102	0.003221463
3	Notch signaling pathway	7.073	3/47	0.006843371
4	Pancreatic cancer	4.448	3/72	0.06375221
5	Maturity onset diabetes of the young	3.952	1/24	0.095158775
6	Phosphatidylinositol signaling system	3.884	2/76	0.100456001
7	Complement and coagulation cascades	3.841	3/69	0.103946526
8	mTOR signaling pathway	3.652	2/52	0.120669151
9	Acute myeloid leukemia	3.448	2/59	0.141487282
10	Systemic lupus erythematosus	3.332	2/144	0.15474593
11	VEGF signaling pathway	3.126	2/74	0.181102595

**Fig. 3:**
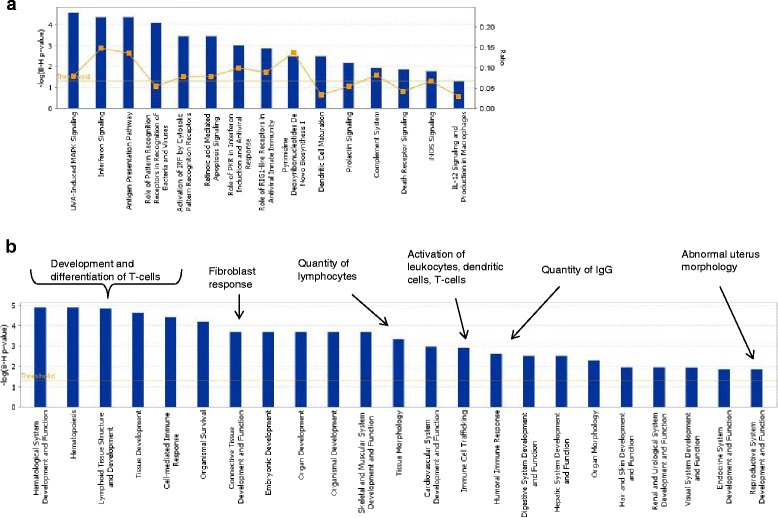
Ingenuity Pathway Analysis (IPA) of genes responding to IBV infection. **a** Canonical biological pathways which are activated in the host upon IBV infection (*p* < 0.05). The line represents the ratio of genes represented within each pathway. **b** The most highly represented (*p* < 0.05) physiological functions of genes differentially expressed during the host response to IBV (in the trachea in susceptible birds (Line 15I)). Specific functions within groups are highlighted

Use of Ingenuity Pathway Analysis (IPA) software also allowed us to determine which biological systems are active during the host response. Up-regulated genes are seen to be part of the canonical biological pathways shown in Fig. 3a. Biological processes involving pattern recognition receptors and interferon signalling feature heavily. The interferon response is a powerful antiviral mechanism, which has previously been shown to be involved in the host response after IBV infection. A very early induction of IFN-γ has been reported in splenocytes [40], and in peripheral blood mononuclear cells (PBMCs) and lung leukocytes [41]. *IFNB* expression has also been reported in trachea between 1 and 2 dpi [42]. We do not see this increase in expression of interferon genes (due to the absence of data earlier than 2 dpi), but we do see the downstream effects, with increased expression of many interferon-induced genes. Specific physiological processes activated upon IBV infection can also be seen in Fig. 3b. The stimulation of various different immune cells is seen along with the indication of reproductive abnormality, which would reflect the problems seen with egg-laying upon IBV infection.

**Fig. 4:**
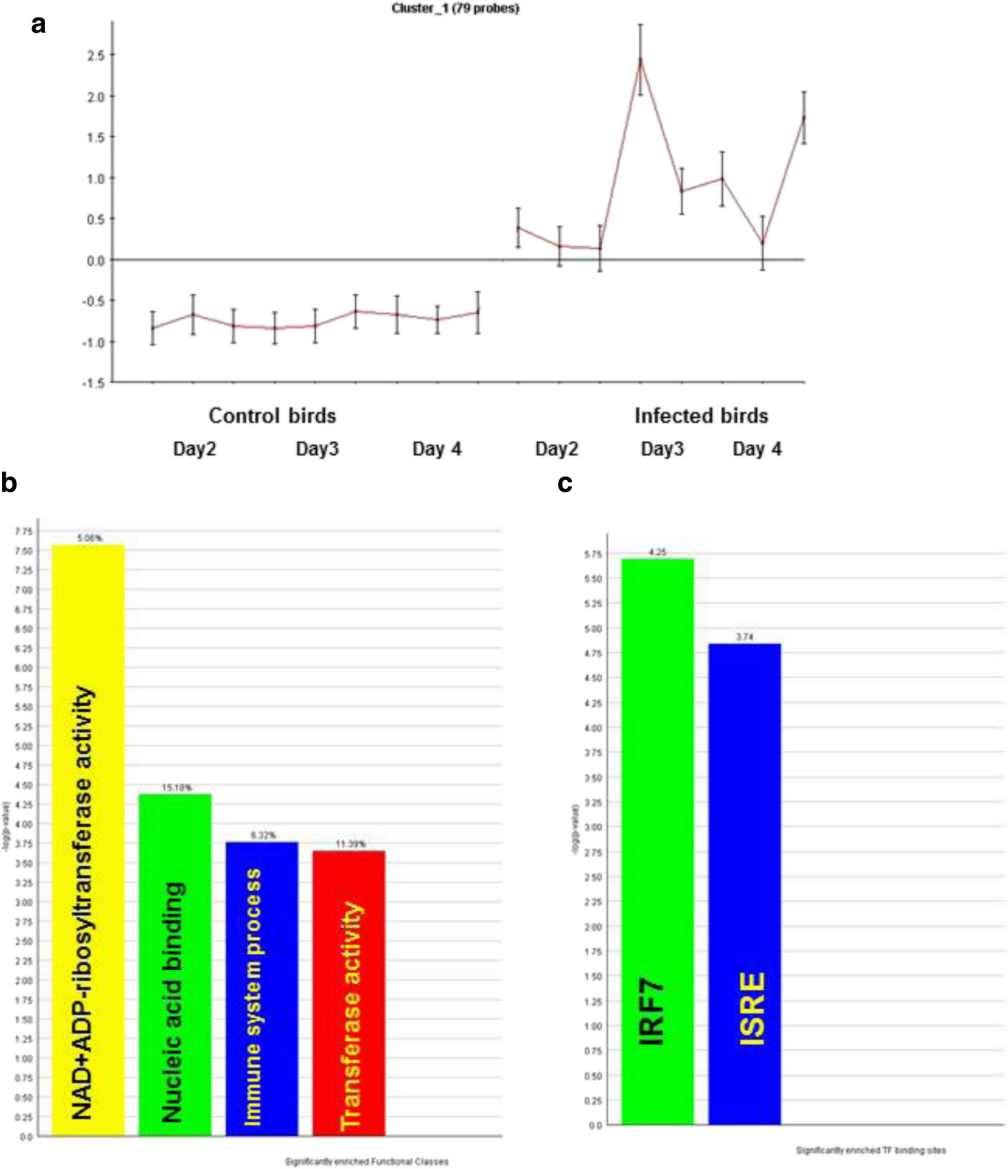
Gene expression cluster analysis of the host response in susceptible birds (Line 15I) using the Expander program (http://acgt.cs.tau.ac.il/expander/expander.html). **a** The expression profile of genes up-regulated during the response to virus. **b** The GO biological process terms which are significantly enriched during the host response to infection. The frequency of genes of a functional class within the examined set is described as a percentage. **c** Binding sites for the transcription factors IRF7 and ISRE are seen to be significantly over-represented in genes up-regulated during the host response to IBV infection. The frequency ratio (frequency in set divided by frequency in background) is shown

In order to cluster genes seen to be involved in the host response to infectious bronchitis into groups with similar expression profiles and probably sharing similar functions or gene regulatory pathways, we utilised the CLICK algorithm within the Expander program [43]. Figure 4a shows the expression profile of genes up-regulated during the response to virus. The Expander program was also used to analyse the Gene Ontology (GO) functional annotations of the genes being differentially expressed. Figure 4b shows the biological process terms, which are significantly enriched in the genes responding during the host response to infection. As would be expected, these include terms like ‘innate immune response’ and ‘antigen processing and presentation’. ‘NAD + ADP-ribosyltransferase activity’ and ‘phosphoinositide binding’ are also highlighted. Transcription factor binding sites present in DE genes which are significantly over-represented were also predicted. Figure 4c shows that genes up-regulated during the host response have a high proportion of *IRF7* and ISRE binding sites. *IRF7* is a transcriptional activator, which binds to the interferon-stimulated response element (ISRE) in IFN promoters and functions as a molecular switch for antiviral activity.

### Differences between susceptible and resistant lines

Analysis of the gene expression differences between infected and control birds across the two lines has provided us with information on how these lines differ in their response to infection. Examination of the gene expression profiles in the control birds of the two different lines also allowed us to identify genes, which are inherently different between the susceptible and resistant birds. It can be seen that there are numerous genes, which show large expression differences between the two lines, even before infection. Dramatic differences in gene expression of certain genes, including *DDT*, *SRI*, *BLB1*, *HSCB*, *BF1*, *BF2*, *SUCLG2, MX1* and *SRI,* which are more highly expressed in the resistant N line compared to the susceptible 15I line are noted (Additional file 2: Table S2 shows all 1930 DE probes) So, it can be seen that these are genes which have inherently different expression levels between susceptible and resistant birds, even before infection occurs. We therefore postulate that some of these genes may play an important role in disease resistance. The potential interactome of IBV has recently been investigated by stable isotope labelling with amino acids in cell culture (SILAC) coupled to a green fluorescent protein-nanotrap pull-down methodology [44]. Host proteins, which bind to the IBV N protein were identified, some of the genes for which, we see as being inherently expressed at higher levels in susceptible birds in this study. These genes include *MYH9*, *CAPRIN1*, *DHX57*, *HNRNPH3*, *RPL27A*, *FMR1*, *C22orf28*, *HNRPDL*, *SFRS3*, *RPL31*, *NPM1* and *RPSA*. This may therefore be one of the reasons why Line 15I is more susceptible to IBV infection – there are more host proteins to which the virus binds, compared with the resistant Line N.

Upon infection, differences in response are also seen between the two lines. Interestingly, apart from *CD38* and *CD4* at 3 dpi and *FKBP5* at 4 dpi, all other differential gene expression between the lines is seen at 2 dpi in this study (Additional file 3: Table S3). CD38 is a glycoprotein found on the surface of many immune cells including CD4+, CD8+, B lymphocytes and natural killer cells and is a marker of cell activation. It functions in cell adhesion, signal transduction and calcium signalling. CD4 is found on the surface of immune cells such as T helper cells, monocytes, macrophages and dendritic cells. It is a membrane glycoprotein which interacts with MHCII antigens. The protein functions to initiate or augment the early phase of T-cell activation. The protein encoded by *FKBP5* is a member of the immunophilin protein family, which play a role in immuno-regulation and basic cellular processes involving protein folding and trafficking. Early defence by the host is a key mechanism for combatting viral infection, and induction of *IFNB* and other innate genes in response to IBV infection has been shown to peak around 18–36 hr post infection [42].

In this study, genes more highly expressed (or less down-regulated) in the resistant N line at 2 dpi include a number of collagen genes (*COL3A1*, *COL1A2*, *COL9A1*, *COL9A2*, *COL6A1* and *COL4A1*) and other genes such as *ACAN*, *FSTL1*, *COMP*, *EIF3A*, *STAT3* and *IGFBP5*. Genes seen to be more highly expressed (or less down-regulated) in the susceptible 15I line include *RBM39*, *MAFB*, *NNK2*, *CCN1*, *MGAT5* and *THRAP3*. One consequence of IBV infection is the production of poor quality, misshapen eggs by infected birds [45]. Some of the genes previously identified as being important for the creation of a healthy eggshell are seen to be more highly expressed by the resistant N line birds after infection in this study. These genes include *COL1A2*, *CRELD2*, *HSP90B1*, *P4HB* and *ERP29* [46]. For a full list of genes differentially expressed between the two lines in trachea (409 DE probes) see Additional file 3: Table S3.

**Fig. 5:**
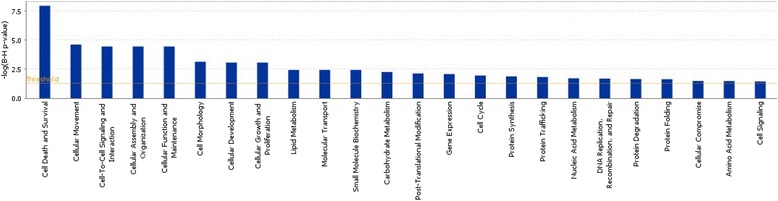
Ingenuity Pathway Analysis (IPA) of genes showing inherent differential expression between susceptible and resistant control birds. This graph shows the most highly represented (*p* < 0.05) molecular functions of DE genes

**Fig. 6:**
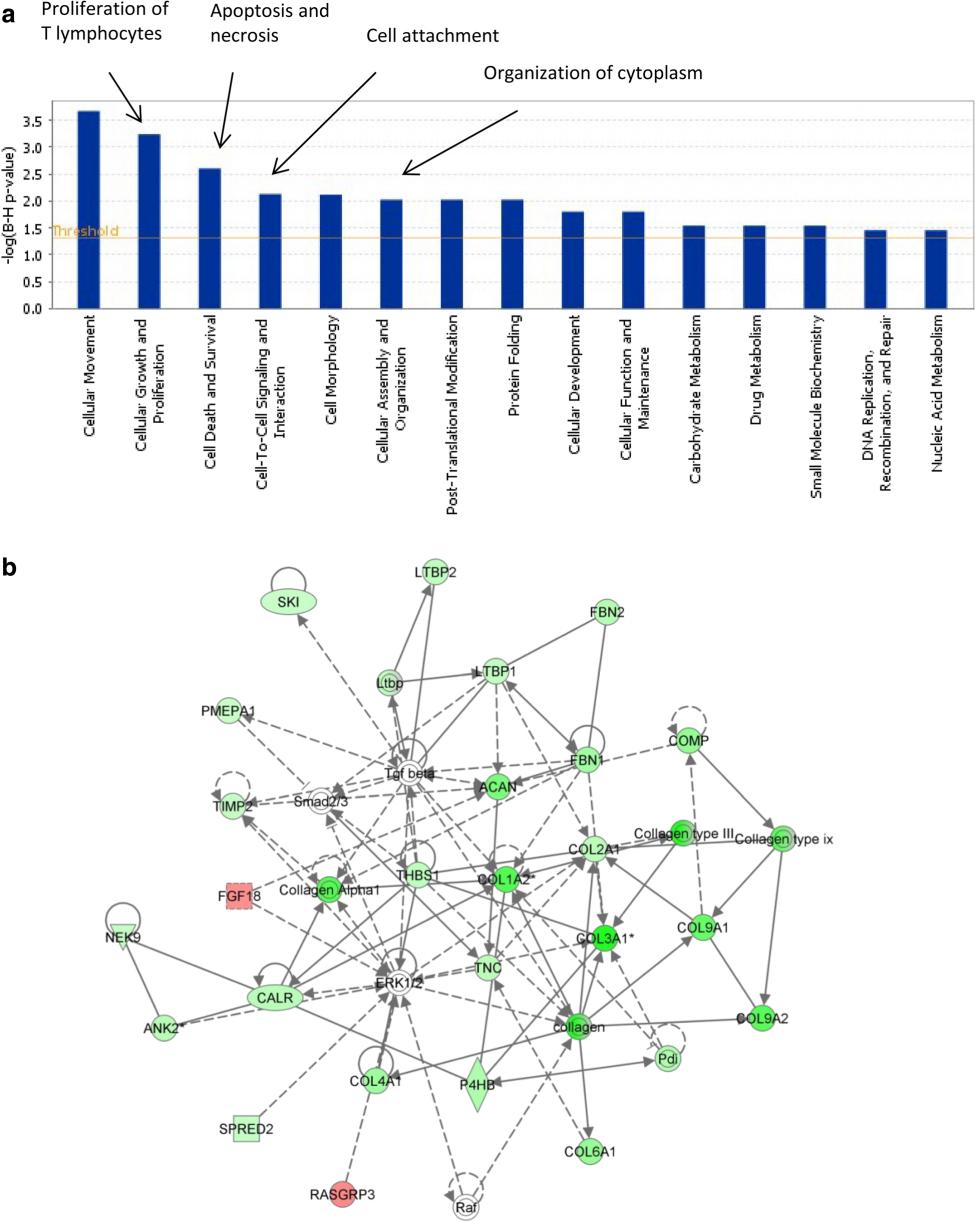
Ingenuity Pathway Analysis (IPA) of genes showing differential expression between susceptible and resistant lines during the host response to IBV infection. **a** The most highly represented (*p* < 0.05) molecular functions of DE genes. **b** This biological network shows genes associated with connective tissue disorders. Genes shown in red are more highly expressed in the resistant line and those in green have higher expression in the susceptible line

IPA analysis of genes showing different inherent expression between lines 15I and N shows that the molecular functions of these genes is primarily concerned with their involvement in cell death and cell adhesion (Fig. 5), two processes previously shown to be significant in infected kidneys [47]. When the differential host responses to infection are examined, it is seen that genes involved in proliferation of T-lymphocytes and genes concerned with cell attachment and cytoplasmic organization are more highly expressed in the resistant line N. Other processes significantly involved are apoptosis and necrosis (Fig. 6a), which have been previously documented in IBV-infected Vero cells by Liu et al. [48]. One of the most perturbed biological networks noted in this analysis is that involving genes related to connective tissue disorders and involve many collagen genes. These genes are more highly expressed in susceptible line 15I birds compared to resistant line N birds (Fig. 6b) suggesting that IBV infection might cause more disorder of eggshell formation in this line [49]. The production of poor quality eggs by IBV infected birds may, in part be a reflection of the expression of these kinds of gene networks compared to that seen in resistant birds.

### Confirmation of differential gene expression by quantitative real-time PCR

Twenty-one genes were selected for qRT-PCR validation (Table 3). These genes were chosen based on their involvement in the host response and whether they were differentially expressed between the susceptible and resistant lines (either inherently or during the course of infection). Of the 21 genes tested, 19 showed comparable differential expression to that determined by the arrays. However, the results for *IFNAR2* and *IGFBP5* were not confirmed (Additional file 4: Figure S1).

**Table 3:** Genes analyzed by qRT-PCR

Gene	Description	Fold change	GenBank	Ensembl
MX1	Interferon-induced GTP-binding protein Mx	6–20	NM_204609	ENSGALG00000016142
C1S	complement component 1, s subcomponent	2–4	NM_001030777	ENSGALG00000014603
IRF7	Interferon regulatory factor 7 (IRF-7)	4–5	NM_205372	ENSGALG00000014297
TLR3	Toll-like receptor 3	4	NM_001011691	ENSGALG00000013468
CCLi7	Chemokine ah221	2–12	AY037860	ENSGALG00000002343
CD38	ADP-ribosyl cyclase 1	4–9	XM_420774	ENSGALG00000014508
FKBP5	FK-506 binding protein 51	5–6	NM_001005431	ENSGALG00000000947
IGFBP5	Insulin-like growth factor binding protein 5	1	XM_422069	ENSGALG00000011468
DDT	D-dopachrome tautomerase	2–3	NM_001030667	ENSGALG00000006350
SRI	sorcin	2	NM_001080865	ENSGALG00000008985
CLU	clusterin	2	NM_204900	ENSGALG00000016587
COX11	Cytochrome c oxidase assembly protein COX11, mitochondrial precursor	2	XM_001233972	ENSGALG00000003017
MMD2	Monocyte to macrophage differentiation factor 2	2	XM_414787	ENSGALG00000004530
IFNAR2	interferon alpha/beta receptor 2	2–3	NM_204858	ENSGALG00000015938
TNFAIP1	tumor necrosis factor, alpha-induced protein 1 (endothelial)	2	NM_001030726	ENSGALG00000005715
MAP4K4	mitogen-activated protein kinase kinase kinase kinase 4	2	NM_001031126	ENSGALG00000008970
MAFK	Transcription factor MafK.	2	NM_204756	ENSGALG00000004189
CCL13	similar to Small inducible cytokine A13 precursor (CCL13)	3	XM_415779	ENSGALG00000024470
TPD52L1	Tumor protein D53 homolog	13–23	NM_204215	ENSGALG00000014843
HSCB	Co-chaperone protein HscB, mitochondrial precursor (Hsc20)	2	XR_026662	ENSGALG00000005706
SUCLG2	succinate-CoA ligase, GDP-forming, beta subunit	2	NM_001006141	ENSGALG00000007652

### Potential candidate genes for IBV resistance

Besides knowing that the MHC B locus has a bearing on disease resistance, the lack of any genetic information or identified QTL meant that we had to rely upon the gene expression differences we saw between susceptible and resistant lines to give us clues as to genes potentially involved in resistance to IBV infection. Identifying genes which were expressed at different levels in the two lines of birds highlighted B-locus genes (*BLB1, BF1, BF2, B-G*) as well as bringing to our attention various other non-MHC genes which, due to their known biology, could be candidates for being involved in resistance to IBV infection (Table 4).

**Table 4:** Potential candidate genes for involvement in resistance to IBV

Gene	Description	Fold change	GenBank	Ensembl
MX1	Interferon-induced GTP-binding protein Mx.	4–24^a^	NM_204609	ENSGALG00000016142
C1S	Complement component 1, s subcomponent	2^a^	NM_001030777	ENSGALG00000014603
IRF7	Interferon regulatory factor 7 (IRF-7).	6^a^	NM_205372	ENSGALG00000014297
TLR3	Toll-like receptor 3	4^a^	NM_001011691	ENSGALG00000013468
C1R	Complement component 1, r subcomponent	2^a^	XM_416518	ENSGALG00000014659
CCLi7	Chemokine ah221	8^a^	AY037860	ENSGALG00000002343
ISG12-2	Interferon stimulated gene 12-2	16–18^a^	NM_001001296	ENSGALG00000013575
IFITM3	Interferon induced transmembrane protein 3	4–5^a^	KC876032	ENSGALG00000004243
CD38	ADP-ribosyl cyclase 1	4^b^	XM_420774	ENSGALG00000014508
CD4	CD4 protein	4^b^	NM_204649	ENSGALG00000014477
FKBP5	FK-506 binding protein 51	2^b^	NM_001005431	ENSGALG00000000947
STAT3	Signal transducer and activator of transcription 3 (acute-phase response factor)	3^b^	NM_001030931	ENSGALG00000003267
OASL	2'-5'-oligoadenylate synthetase-like	4^c^	NM_205041	ENSGALG00000013723
DDT	D-dopachrome tautomerase	62–72^c^	NM_001030667	ENSGALG00000006350
SRI	Sorcin	65–89^c^	NM_001080865	ENSGALG00000008985
BLB1	MHC Class II beta 1 and 2 domains	10–22^c^	NM_001044694	ENSGALG00000000141
IFNAR2	Interferon alpha/beta receptor 2	2–3^c^	NM_204858	ENSGALG00000015938
TPD52L1	Tumor protein D53 homolog	12–14^c^	NM_204215	ENSGALG00000014843
BCL2L1	BCL2-like - apoptosis regulator	1.7^c^	NM_001025304	ENSGALG00000006211
FAIM2	Fas apoptotic inhibitory molecule 2	1.7^c^	XM_004950568	ENSGALG00000027555
CIAPIN1	Cytokine Induced Apoptosis Inhibitor 11	1.7^c^	NM_001005834	ENSGALG00000005706
HSCB	Co-chaperone protein HscB, mitochondrial precursor (Hsc20).	8–10^c^	XR_026662	ENSGALG00000005706
BF1	MHC class I antigen B-F minor heavy chain	13–27^c^	NM_001097530	ENSGALG00000000178
BF2	Major class I glycoprotein precursor	7–8^c^	NM_001031338	ENSGALG00000000178
SUCLG2	succinate-CoA ligase, GDP-forming, beta subunit	16–32^c^	NM_001006141	ENSGALG00000007652

^a^Upregulated in response to infection in the susceptible line

^b^Higher expression in response to infection in the resistant than in the susceptible line

^c^Inherently higher expression in the resistant line

*MX1*, *C1S*, *IRF7*, *TLR3*, *C1R*, *CCLi7*, *ISG12-2* and *IFITM3* are all strongly induced during the host response to IBV infection. They are all innate immune genes which could potentially have a role in determining susceptibility to the virus. MX1 and IFITM3 are already established as anti-viral molecules [50–52]. *CD38*, *CD4*, *FKBP5* and *STAT3* all show a higher level of expression during the host response in the resistant birds compared to that of the susceptible birds, indicating their involvement in the host defence mechanism. CD38 and CD4, with their role as receptors on immune cells, as described above, are obvious candidates, along with FKBP5 as an immune-regulator. *STAT3* is activated by various cytokines and growth factors and functions in cellular processes such as cell growth and apoptosis.

Even before infection, many genes are seen to be highly differentially expressed between lines 15I and N. OASL is an interferon-induced molecule known to have anti-viral activity against certain viruses such as hepatitis C virus. DDT is highly homologous to the macrophage migration inhibition factor, MIF. We have also shown it to be highly differentially expressed in other chicken lines, which are susceptible or resistant to Marek’s Disease virus [53]. IFNAR2 is an obvious candidate prediction, as the interferon response is central to the host’s defence against IBV infection. *TPD52L1*, *BCL2L1*, *FAIM2* and *CIAPIN1* are all known to be involved in regulation of apoptosis, a process seen to be important during IBV infection. *HSCB*, *SRI*, and *SUCLG2*, although not having an obvious potential biological role in disease resistance, are highly differentially expressed between susceptible and resistant lines and should thus be considered as potential candidates.

## Conclusions

Resistance to IBV infection is brought about by the immune response after the virus has entered the host and is not due to prevention of initial viral infection. There is a small initial innate response at 2 dpi, with much more gene expression seen at 3 and 4 dpi. Analysis of genes being activated or inhibited upon infection shows that the biological pathways primarily affected during IBV infection include MAPK signalling, those involved in the interferon response and those involving pattern recognition receptors.

Susceptible and resistant lines show a differential host response mostly at 2 dpi. There are also genes which are inherently different between the two lines studied, including many genes, which control the apoptotic potential of the host. These differences seen in gene expression levels, allow us to postulate on many candidate genes for disease resistance. Some potential candidates for involvement in disease resistance include genes already known to confer resistance to other viral infections (*MHC-B* locus genes, *MX1*, *OASL* and *IFITM3*), genes involved in apoptotic processes (*TPD52L1*, *BCL2L1*, *FAIM2* and *CIAPIN1*) and others which could be potential candidates due to their known biology (e.g. *DDT* and *CD4*).

## Availability of supporting data

Array data have been submitted to Array Express (http://www.ebi.ac.uk/arrayexpress/) under the accession number E-TABM-1128.

## Additional files

Additional file 1: Table S1. Gene expression seen during the host response to IBV infection in the trachea of susceptible birds. (XLSX 24 kb) Additional file 2: Table S2. Gene expression differences found to be inherent between susceptible and resistant lines in the trachea. (XLS 386 kb) Additional file 3: Table S3. Genes found to be differentially expressed between susceptible and resistant lines in response to IBV infection in the trachea. (XLS 107 kb) Additional file 4: Figure S1. qRT-PCR analysis of 21 genes differentially expressed during IBV infection. (**A**). MX1 (**B**). C1S (**C**). IRF7 (**D**). TLR3 (**E**). CCLi7 (**F**). DDT (**G**). SRI (**H**). CLU (**I**). COX11 (**J**). IFNAR2 (**K**). TNFAIP1 (**L**). TP-D53 (**M**). MAP4K4 (**N**). MAFK (**O**). CCL13 (**P**). HSC20 (**Q**). SUCLG2 (**R**). MMD2 (**S**). CD38 (**T**). FK506-BP51 (**U**). IGFBP5. Graphs A-E show expression changes during the host response in the susceptible line. Graphs F-R show inherent differences in gene expression between susceptible and resistant control birds, while graphs S-U indicate differential gene expression in susceptible and resistant lines during the host response. (PDF 97 kb)

## Abbreviations

BSA: Bovine serum albumin
CID_50_: mean chicken infectious dose
DE: Differentially expressed
FARMS: Factor Analysis for Robust Microarray Summarization
FDR: False discovery rate
GCOS: GeneChip Operating Software
GO: Gene ontology
IB: Infectious bronchitis
IBV: Infectious bronchitis virus
KEGG: Kyoto Encyclopaedia of Genes and Genomes
MHC: Major histocompatibility complex
OD: Optical density
PBS: Phosphate buffered saline
PLIER: Probe Logarithmic Error Intensity Estimate
qRT-PCR: Quantitative reverse transcription polymerase chain reaction
QTL: Quantitative trait loci
RIN: RNA integrity number
SARS: Severe acute respiratory syndrome
SILAC: Stable isotope labelling with amino acids in cell culture
TFBS: Transcription factor binding site
