# Supplementary material for: The early immune response to infection of chickens with Infectious Bronchitis Virus (IBV) in susceptible and resistant birds
Source: BMC Vet Res. 2015 Oct 9;11:256. doi: 10.1186/s12917-015-0575-6 (PMC4600211; doi:10.1186/s12917-015-0575-6)
Supplement: Additional file 4: Figure S1. — qRT-PCR analysis of 21 genes differentially expressed during IBV infection. (A). MX1 (B). C1S (C). IRF7 (D). TLR3 (E). CCLi7 (F). DDT (G). SRI (H). CLU (I). COX11 (J). IFNAR2 (K). TNFAIP1 (L). TP-D53 (M). MAP4K4 (N). MAFK (O). CCL13 (P). HSC20 (Q). SUCLG2 (R). MMD2 (S). CD38 (T). FK506-BP51 (U). IGFBP5. Graphs A-E show expression changes during the host response in the susceptible line. Graphs F-R show inherent differences in gene expression between susceptible and resistant control birds, while graphs S-U indicate differential gene expression in susceptible and resistant lines during the host response. (PDF 97 kb) [file 12917_2015_575_MOESM4_ESM.pdf]

Supplementary Figure 1

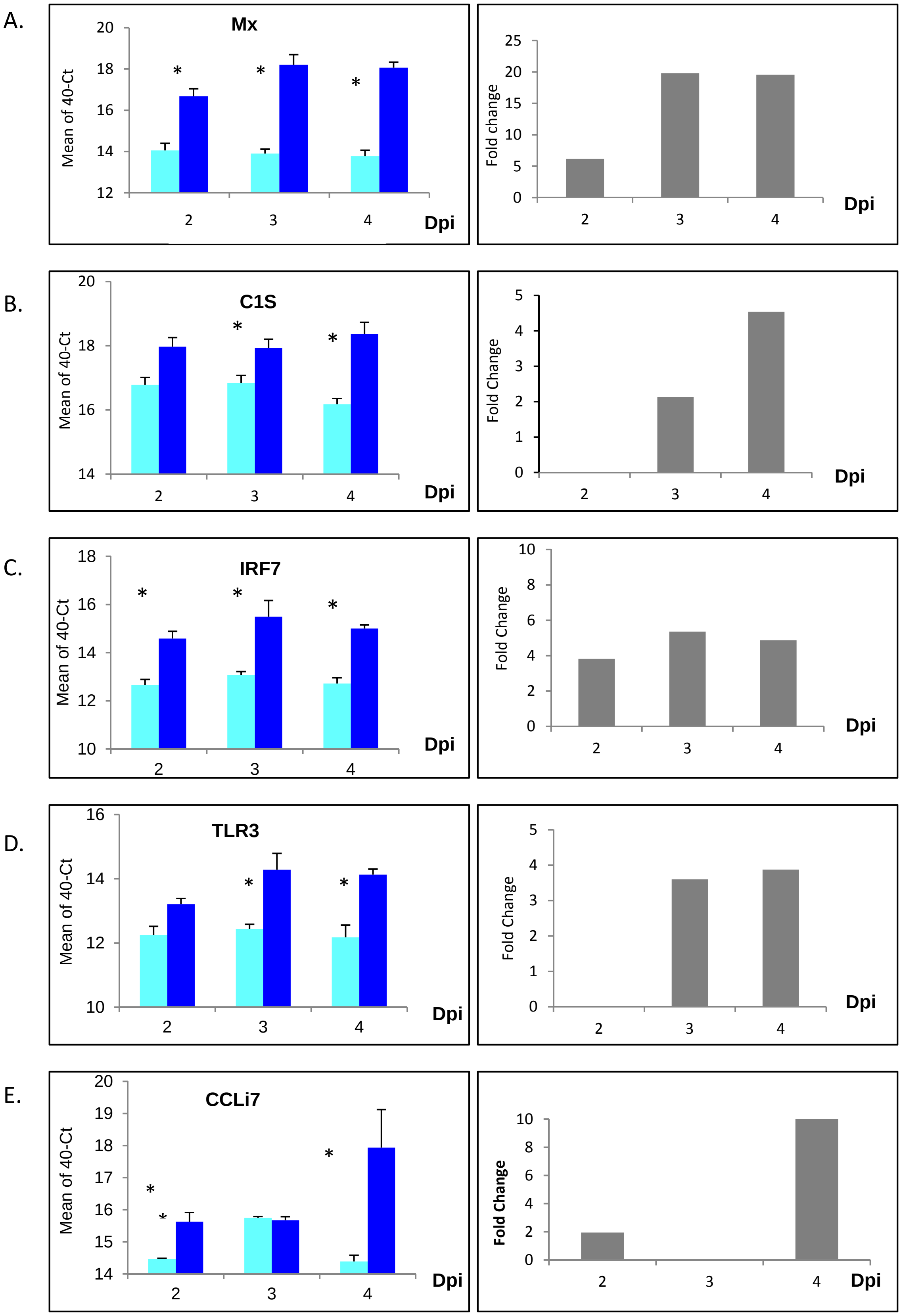

F.

G.

H.

I.

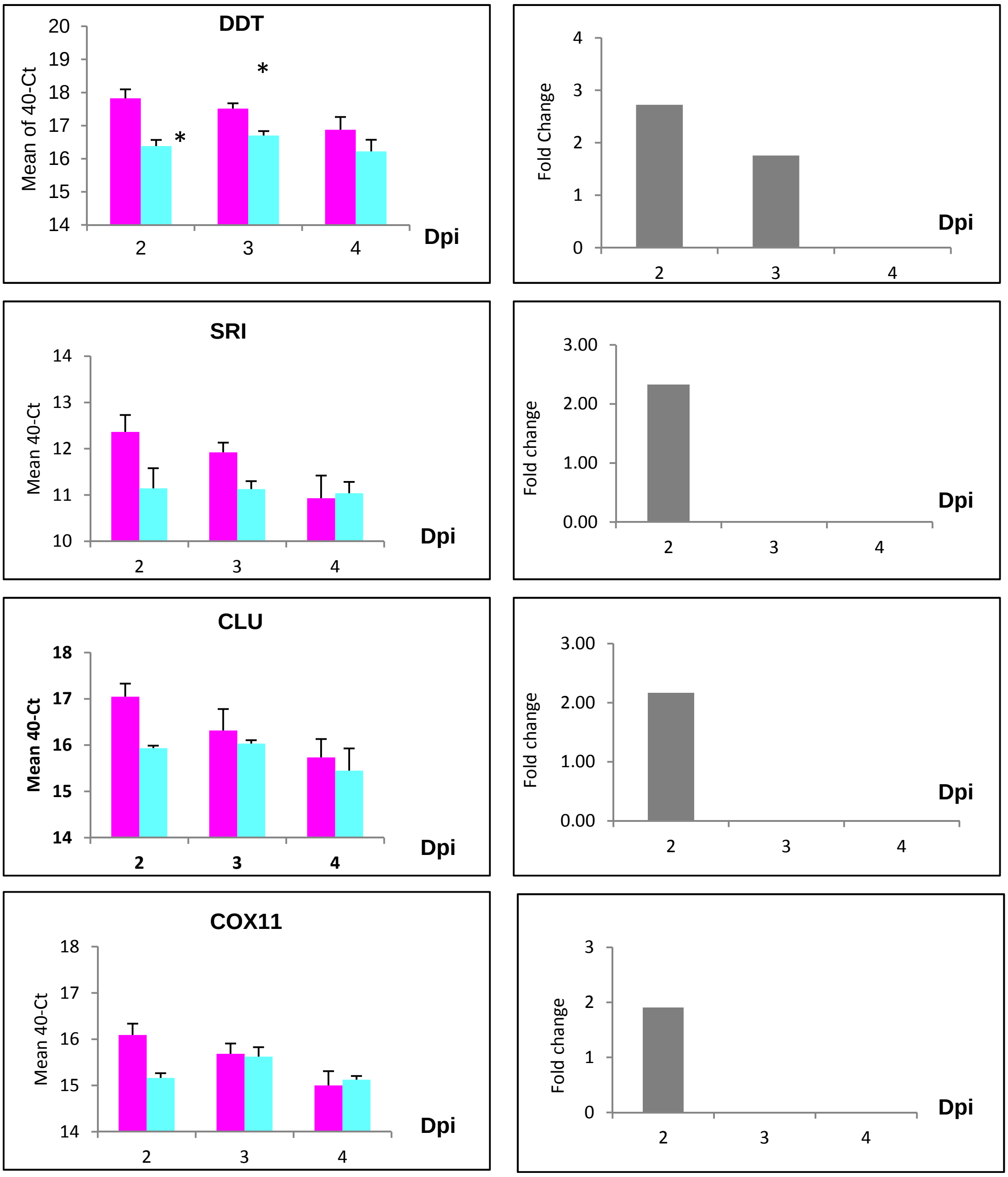
DDT
Mean of 40-Ct
20
19
18
17
16
15
14
*
*
2
3
4
Dpi
Fold Change
4
3
2
1
0
2
3
4
Dpi
SRI
Mean 40-Ct
14
13
12
11
10
2
3
4
Dpi
Fold change
3.00
2.00
1.00
0.00
2
3
4
Dpi
CLU
Mean 40-Ct
18
17
16
15
14
2
3
4
Dpi
Fold change
3.00
2.00
1.00
0.00
2
3
4
Dpi
COX11
Mean 40-Ct
18
17
16
15
14
2
3
4
Dpi
Fold change
3
2
1
0
2
3
4
Dpi

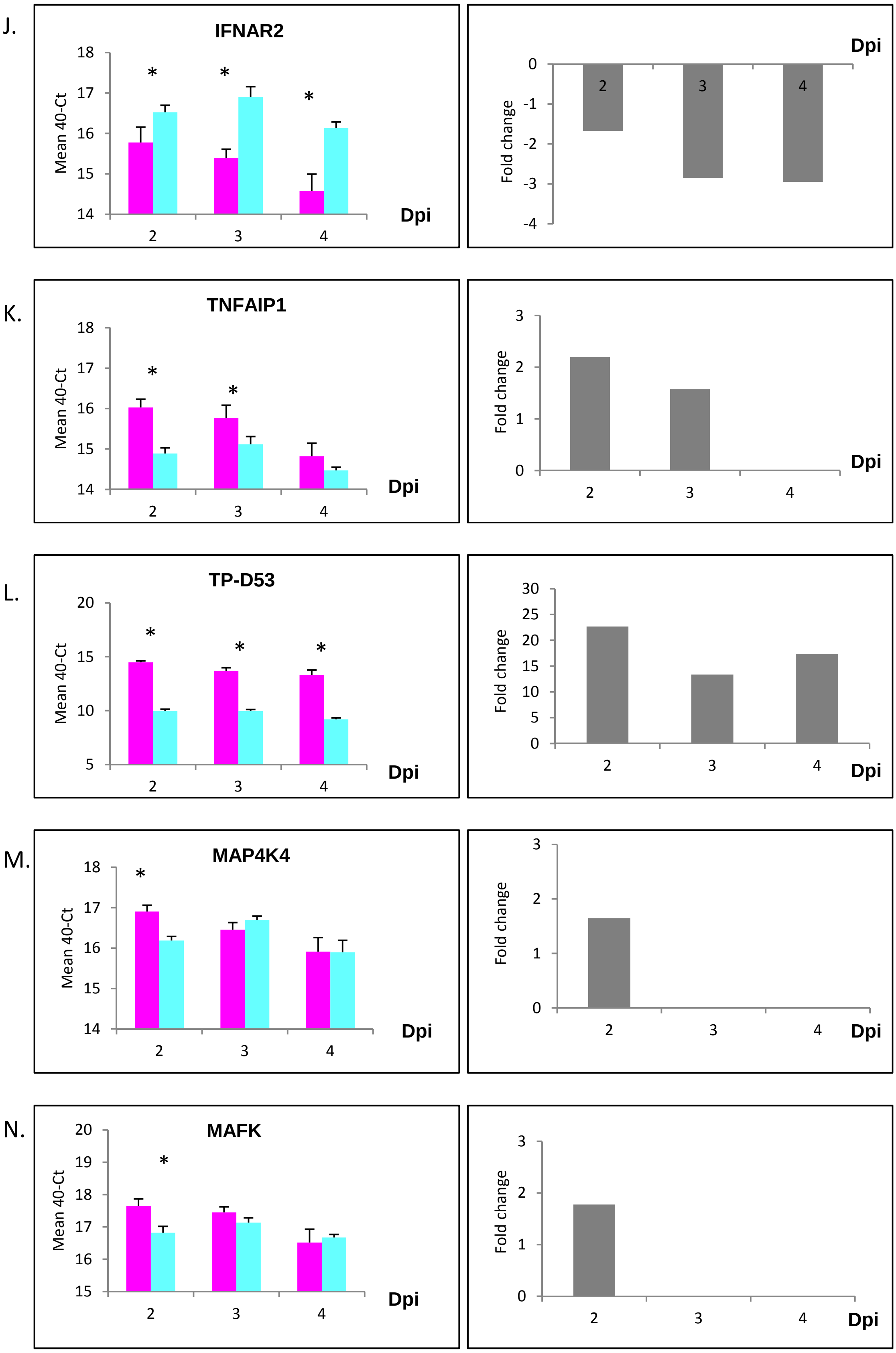

J.
IFNAR2
Mean 40-Ct
18
17
16
15
14
*
*
*
2
3
4
Dpi
Fold change
0
-1
-2
-3
-4
K.
TNFAIP1
L.
TP-D53
20
10
5
30
25
M.
MAP4K4
N.
MAFK
19

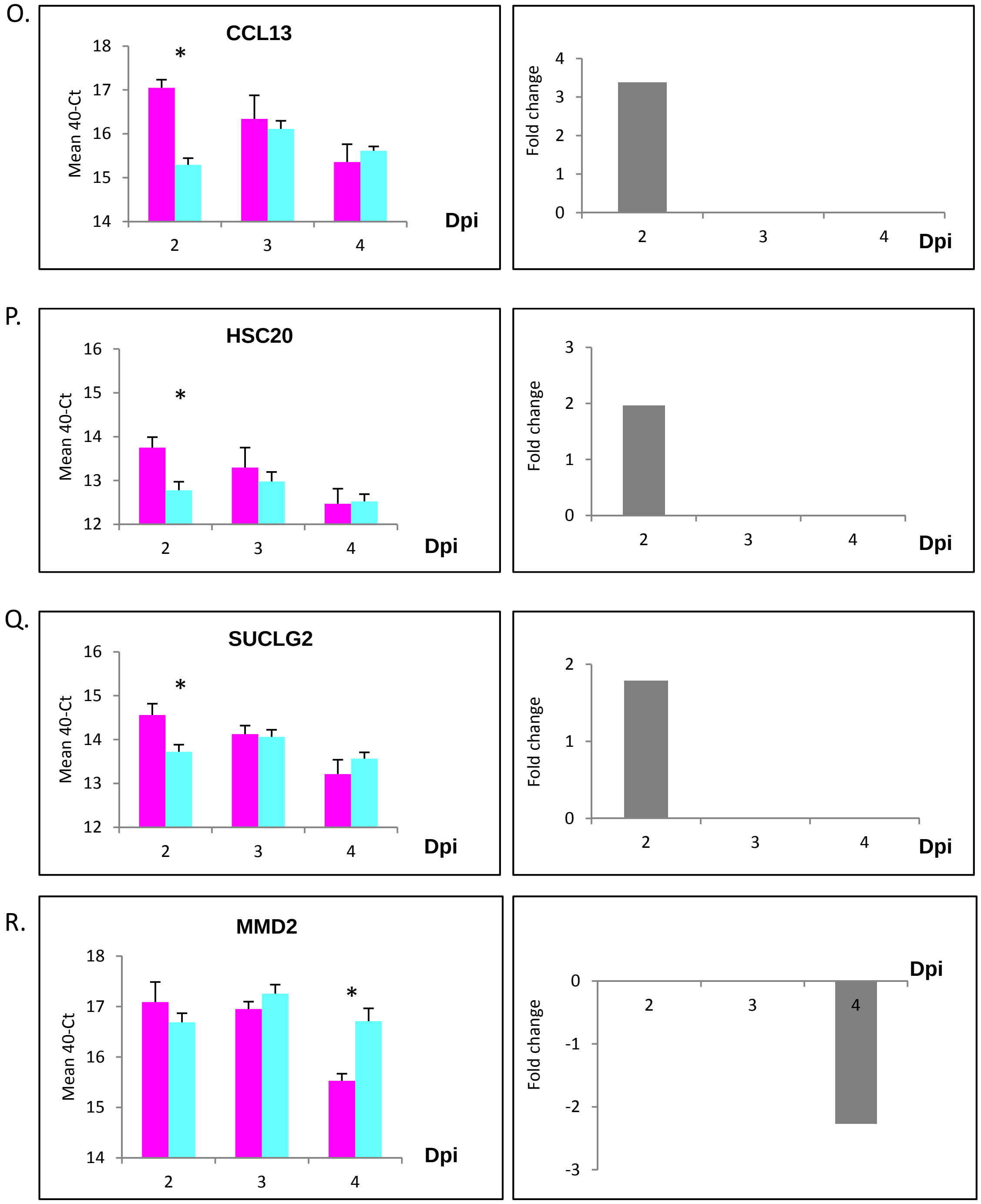

O.
CCL13
Mean 40-Ct
18
17
16
15
14
*
2
3
4
Dpi
Fold change
4
3
2
1
0
2
3
4
Dpi
P.
HSC20
Mean 40-Ct
16
15
14
13
12
*
2
3
4
Dpi
Fold change
3
2
1
0
2
3
4
Dpi
Q.
SUCLG2
Mean 40-Ct
16
15
14
13
12
*
2
3
4
Dpi
Fold change
2
1
0
2
3
4
Dpi
R.
MMD2
Mean 40-Ct
18
17
16
15
14
*
2
3
4
Dpi
Fold change
0
-1
-2
-3
2
3
4
Dpi

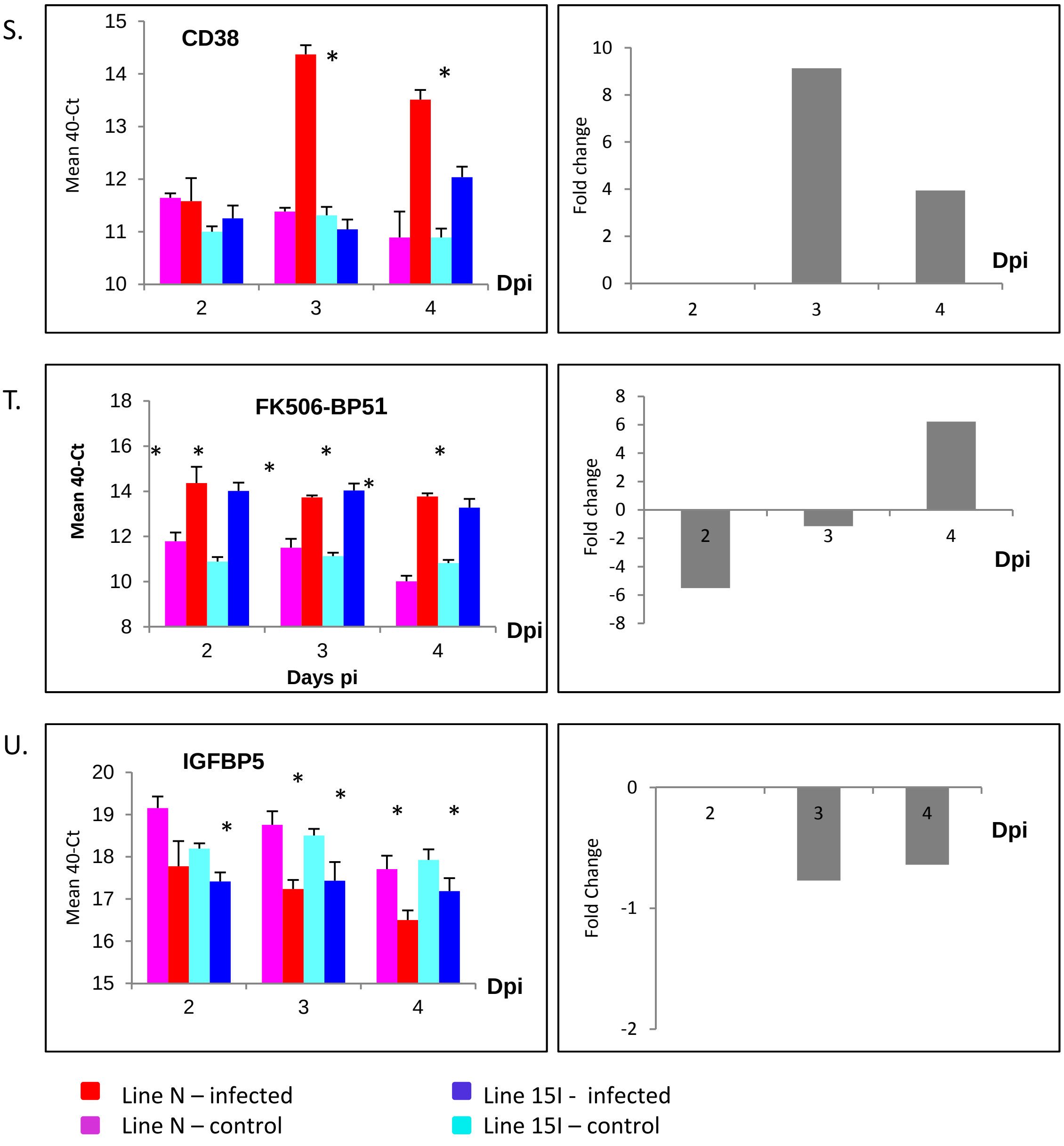

S.
CD38
Mean 40-Ct
15
14
13
12
11
10
2
3
4
Dpi
*
*
Fold change
10
8
6
4
2
0
2
3
4
Dpi
T.
FK506-BP51
Mean 40-Ct
18
16
14
12
10
8
2
3
4
Days pi
Dpi
*
Fold change
8
6
4
2
0
-2
-4
-6
-8
2
3
4
Dpi
U.
IGFBP5
Mean 40-Ct
20
19
18
17
16
15
2
3
4
Dpi
*
Fold Change
0
-1
-2
2
3
4
Dpi
Line N – infected
Line N – control
Line 15I - infected
Line 15I – control
